# Diphtheria

**Published:** 1861-02

**Authors:** R. W. I’Anson

**Affiliations:** Dinwiddie county, Virginia


### *Diphtheria.* By R. W. I'Anson, M. D., of Dinwiddie county, Virginia.

My attention has been drawn particularly to this disease from articles contained in the journals, and from the importance of the disease itself. During the fall and winter of 1854, it prevailed in Chesterfield county to a considerable extent, and some thirty cases came under my notice. All of these were successfully treated with the exception of one, pnuemonia ensued in this and soon terminated its existence. As noticed then, there seemed to be close affinity between diphtheria and scarlatina. Early in the fall the disease was diagnosed as diphtheria ; there was no eruption, nor did the fever run high, but toward the beginning of winter eruption was visible and very high fever, so that scarlet fever actually made its appearance. The same disease prevails to a limited extent where I am at this time, but no eruption attends it. What may be the effect of *cold* upon its character, I am not prepared to say. Diphtheria is spoken of as being a reeent disease, at least such seems to be the opinion entertained by some old practitioners, but such is not the case, for it would be no hard task to adduce testimony of its having prevailed epidemically, from the time of Cullen, (for he describes the disease, eruption and all, under the head of cynanche maligna,) in other countries, and also in various portions of the United States. It is also looked upon as being a very fatal disease. This may be the result of several causes : first, either because treated too actively *generally*, or not active enough *locally ;* or second, from neglect. If all diseases tended as far as this to prove the power and efficacy of medicine, there would remain no doubts of its utility on the mind of any. It is simply a disease, the course of which is rapid, and, like all others of that class, should be taken in time—*obsta-principiis* is as true in this as in any other instance. So far as my experience has gone in the management of this affection, the general treatment should be simple ; *i. e.*, an avoidance of every thing *harsh or drastic.*—

The fever is always of an *asthenic* or *typhous* character, and from the degree of prostration which attends it, shows the system to be primarily effected, or, at any rate, very early influenced by the mobific cause. In the beginning, an emetic of salt and water given warm, or ipecacuanha, will produce a good influence on the secretions of the throat; and throughout the attack, whenever there is much secretion lodged in the throat, emetics may be used with great relief. After emesis, the bowels should be opened by oil and spirits of turpentine. A stimulating and supporting plan of treatment should now be adopted, such as camphor and quinine —1 to 5 grs. each—with ipecac, avoiding the preparations of opium on account of the disposition to stupor, using the ext. hyosciamus in its stead. Capsicum, used in combination with the powder of camphor and quinine, will be found an excellent remedy. The bowels should be opened with rhubarb, or some mild aperient. Diet, nutritious, and in feeble cases, stimulating. There is a tendency toward pneumonia, of course it will be typhoid, small doses of calomel or blue mass must be added to the powder, and given regularly every four hours, until the gums are slightly touched; but the main reliance must be placed in *repeated* blistering, both in front and behind the chest. At this stage great advantage will be derived from the use of spirits of turpentine, together with stimulating expectorants, as decoction of seneka and syrup of squills.

*Local Treatment.*—This is of the utmost importance, but no apprehensions of danger on the part of the patient, or friends, or impulsiveness on the part of the practitioner, should impel him to make too *free* use of irritating articles in the form of washes and gargles. In my hands the nitrate of silver, 20 grs. to the ounce of water, has proved a satisfactory remedy, the throat to be thoroughly mopped once a day for three or four days, when the disease will generally yield, then to be reduced to half its strength and continued *pro ne nata.* In the meantime some mild astringent gargle as alum, vinegar and honey in sage tea; or black pepper, salt and vinegar may be used, but none of these should be used oftener than three times a day. Whereas, warm milk and water will always be found to be soothing as well as beneficial to the patient, and may be frequently used.

*External local treatment.*—A pepper poultice is what I generally use; also, flannel annointed with spirits of turpentine. Should the cervical glands remain enlarged during convalescence, the iodide of potasium used internally will be beneficial. Whenever a disease, as insidious, and yet so fatal as the one under consideration, is known to prevail in one's section, it is necessary that the medical man should be ever on the alert. He should inquire into and even examine every case, especially among children, whatever the complaint may be.

Since the above was written, a case has been under my care which it is gratifying to me to report, for out of fifty or sixty cases of this disease, it was the first and only one in which the *croupal* symptom predominated; and furthermore, as showing to the young and inexperienced physician the utter impossibility of following strictly any plan of treatment—even his own; but that he should be prepared to treat the case according to the symptoms presented at the time by the disease.

Nov. 16th, 8 o'clock, A. M.: Called to see a female child, aged five, previously healthy, fleshy. Saturday night previous had a croupal cough, aphonia complete, but not being confined to bed, her parents thought it afflicted with a slight cold, and treated it accordingly. The child grew worse on Friday, when I was sent for. Found her with great difficulty in breathing; chest heaving heavily up and down; but little fever; had never complained of her throat; no external swelling. Upon examination found ten or twelve white patches of false membrane or tonsils. Diagn. diphtheria with croup. Treatment, emetic, ipecac 20 gr., tartar emetic 2 gr., all given during an hour; vomited several times; throat mopped with nitrate silver 20 gr. to ounce and extended into trachea; then cal. 4 gr., ipecac 4 gr., tartar emetic 1 gr., quinine 8 gr. capsicum 8 grs. in 8 powders, one every hour; pepper poultice to throat; left her at 10½ much improved. A gargle of alum, vinegar and honey three times per day.

Saw her at 5 P. M.: Very much improved; can speak a little; had vomited at times; breathes freely and easily; considerable stupor; took all the powders but one; complained of headache. Treatment, cal. 6 grs., ipecac 2 grs.; tartar emetic 1 gr., camph. 8 grs., capsicum 4 grs., divided in 4 powders, one every 4 hours through night. Stimulating bath to feet, and pepper poultice to chest—skin acting well.

17th, morning: Not so well as evening before; cough dry; rested well; bowels open once during night—stool dark. Treatment, emetic, ipecac 20 grs., tartar emetic 3 grs., syrup squills 1 ℥, in seneka tea, ⅛ part every 15 minutes; took all but did not vomit much. Afterwards put her on cal. 8 grs., ipecac 4 grs., camph. 16 grs. ext. hyosiam. 4 grs., capsicum 8 grs., divided in 8 powders, one every 2 hours; after taking two doses, gums being swollen, put off to 4 hours during night. Decoc. seneka and syrup squills, given in 4 hours. Throat mopped twice to-day with solution of nitrate of silver, 20 gr. to ounce. 4 P. M.—Croupal cough, not so dry as in the morning; stupor all the time; had soup twice to-day; air does not enter lungs in front; fever slight; pulse weak; blister in front of chest.

18th, morning: Symptoms entirely relieved; breathes easily; gums sore; change brought about by the combined influence of mercury and the blister—was sudden; bowels opened once—dark and thick; noticed the left nostril almost closed by false membrane; wished to examine the throat, but her father, who fancied himself of the knowing ones, and had frequently given his opinion as to the disease and amount of physic given, positively refused to allow it, and I positively refused to have any thing more to do with the case. When I undertook the case the chances were against me, but diphtheria is more chronic than croup, and the treatment had to be divided between the two diseases. The bowels were never deranged during the whole treatmrnt.—*Maryland & Virginia Medical Journal.*

